# Heterologous Expression, Enzymatic Characterization, and Ameliorative Effects of a Deoxynivalenol (DON)-Degrading Enzyme in a DON-Induced Mouse Model

**DOI:** 10.3390/toxins17120588

**Published:** 2025-12-09

**Authors:** Haorui Zhou, Bingtao Xu, Yuqing Peng, Jiahao Mao, Xuelei Zhang, Yongpeng Guo, Yong Zhang

**Affiliations:** 1 School of Biological Engineering, Henan University of Technology, Zhengzhou 450001, China; haoruizhou@stu.haut.edu.cn (H.Z.); 2021920177@stu.haut.edu.cn (B.X.); 2022930654@stu.haut.edu.cn (Y.P.); 221060500201@stu.haut.edu.cn (J.M.); zhangxuelei1020@126.com (X.Z.); 2 College of Animal Science and Technology, Henan Agricultural University, Zhengzhou 450046, China

**Keywords:** deoxynivalenol (DON), DON-degrading enzyme, heterologous expression, enzyme kinetics, gut microbiota

## Abstract

Deoxynivalenol (DON), a mycotoxin produced by *Fusarium* species, severely contaminates grains and feed, posing a continuous threat to human and livestock health. In this study, the DON-degrading enzyme (DDE) gene from *Devosia* sp. JA3 was heterologously expressed in *Escherichia coli*. Enzyme kinetics revealed that DDE exhibited optimal activity at 37 °C and pH 7.0, with a Km of 0.32 mM and a Vmax of 563.3 nmol/(min·mg). Under optimized conditions, DDE efficiently oxidized DON to 3-keto-DON, achieving a degradation rate of 82.51% within 12 h. Further investigation in C57BL/6J mice showed that oral administration of 2 mg/kg DON significantly reduced antioxidant capacity, caused liver damage, impaired intestinal barrier function, induced intestinal inflammation and apoptosis, and disrupted the gut microbiota. DDE treatment effectively alleviated these DON-induced effects by restoring antioxidant capacity, ameliorating liver injury, downregulating pro-inflammatory and apoptotic genes, upregulating barrier-related genes, and restoring the gut microbiota balance, thereby protecting intestinal health. These findings demonstrate DDE’s excellent DON-degrading capacity and biosafety, providing new technical evidence for DON detoxification applications.

## 1. Introduction

Deoxynivalenol (DON), a type B trichothecene mycotoxin, is a toxic secondary metabolite produced by *Fusarium* species [1]. DON contamination is frequently detected in cereals and their processed by-products, often at levels exceeding regulatory limits [2,3,4,5]. Due to its heat and acid resistance, it is difficult to remove DON during conventional storage and food processing [3,4]. Acute exposure to DON can cause nausea, vomiting, gastroenteritis, diarrhea, and reduced food intake, while chronic exposure may lead to teratogenicity, cytotoxicity, genotoxicity, and immunotoxicity [6,7,8].

To mitigate the harmful effects of DON on humans and animals, researchers have continuously explored various methods for DON detoxification. Physical detoxification methods include heat treatment [9], irradiation [10], and adsorption [11], while chemical detoxification primarily involves the use of chemical reagents and ozone fumigation [12,13]. However, these methods are limited in practical application due to issues such as their low efficiency, the formation of unknown by-products, non-specificity, and nutrient loss [14]. Biological detoxification uses microbial or cell-free enzymes, which convert DON into less toxic or non-toxic metabolites [15]. Among biological detoxification methods, the enzymatic detoxification technique stands out as a promising strategy, owing to its high reproducibility, uniformity, simplicity of operation, and strong safety profile.

A variety of microorganisms with DON-degrading capabilities have been identified to date, with the primary degradation pathways involving the oxidation and epimerization at the C3 hydroxyl group, as well as ring-opening oxidation of the epoxide group at the C12 and C13 positions. In the C3 hydroxylation and epimerization pathway, strains such as *Devosia mutans* 17-2-E-8 [16], *Nocardioides* sp. ZHH-013 [17], and *Pelagibacterium halotolerans* ANSP101 [18] can convert DON into 3-keto-DON or 3-epi-DON, both of which exhibit a significantly lower toxicity than DON itself [19]. In the epoxide ring-opening pathway, the catfish intestinal microbiota C133 [1,20,21], *Slackia* sp. D-G6 [22], and microbiota PGC-3 [23], among others, can catalyze the reaction to produce DOM-1, a metabolite with reduced toxicity [21].

At the molecular level, several key enzymes involved in DON degradation have been identified and characterized. During this reaction, the initial dehydrogenation step converts DON to 3-keto-DON, a reaction typically catalyzed by dehydrogenases that depend on cofactors such as pyrroloquinoline quinone (PQQ) [24,25,26,27,28]. For example, the DepA enzyme, isolated from *Devosia* 17-2-E-8 [29], and the QDDH enzyme from *Devosia* D6-9 [30] both belong to the family of PQQ-dependent dehydrogenases and share a high sequence identity. In contrast, DON dehydrogenase (DDH) [31], which operates independently of PQQ, utilizes electron acceptors such as phenazine methosulfate (PMS) and dichlorophenol indophenol (DCPIP). Sequence alignment reveals that DDH shares a low sequence identity with both DepA and QDDH.

Although several key enzymes involved in DON degradation have been identified and characterized, existing studies have primarily focused on in vitro enzymology, with limited systematic explorations of their in vivo applications. Therefore, in this study, the gene sequence of the DON-degrading enzyme (DDE) was cloned from *Devosia* sp. JA3 and DDE in *Escherichia coli* was heterologously expressed and purified and its enzymatic properties were investigated. Liquid chromatography–mass spectrometry (LC-MS) was used to analyze the degradation products. Finally, the effects of applying DDE in DON-exposed mice were explored, providing comprehensive scientific evidence for the advancement and utilization of DON-degrading enzymes.

## 2. Results and Discussion

### 2.1. Functional Validation of the DON-Degrading Enzyme (DDE)

**Figure 1:**
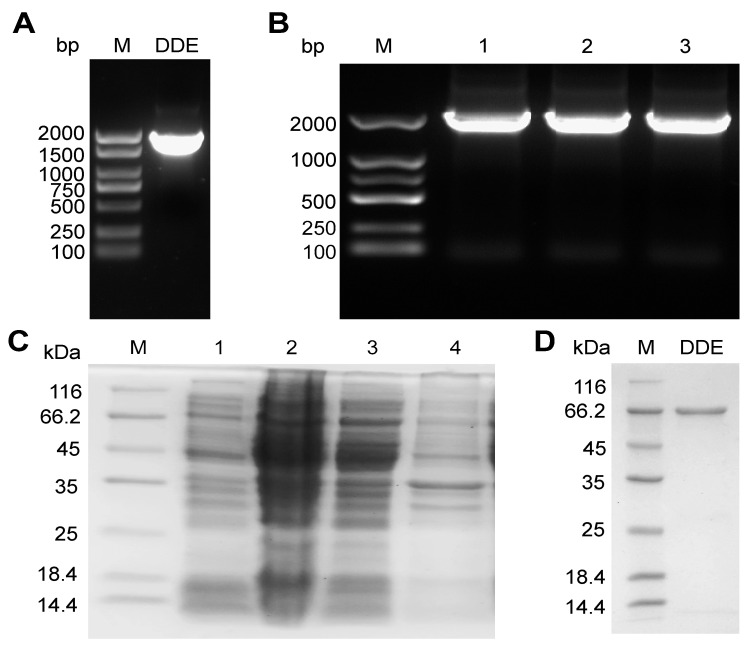
Cloning, expression, and purification of recombinant DDE in *E. coli* BL21(DE3). (**A**) PCR amplification of the DDE gene from *Devosia* sp. JA3 genomic DNA. M: 1 kb DNA ladder. (**B**) Colony PCR verification of positive transformants using T7 primers. M: 1 kb DNA ladder; lanes 1–3: positive clones. (**C**) SDS-PAGE analysis of DDE localization after IPTG induction (0.5 mM, 20 °C, 6 h). M: protein marker (10–180 kDa); 1: whole-cell lysate before induction; 2: whole-cell lysate after induction; 3: supernatant after cell lysis (soluble fraction; DDE primarily localized here); 4: pellet after cell lysis (insoluble fraction). (**D**) SDS-PAGE analysis of purified DDE (Ni-NTA magnetic beads). All experiments were performed in triplicate (*n* = 3); gels shown are representative.

The DON-degrading enzyme (DDE) gene (theoretical length: 1791 bp) was amplified by PCR using the whole-genomic DNA of *Devosia* sp. JA3 as a template. As shown in Figure 1A, the PCR product displayed a single bright band within the 1500–2000 bp range, consistent with the expected size. The DDE gene was ligated into the pET31-b vector and transformed into *E. coli*, and positive clones were selected on LB-Amp plates. PCR verification using T7 universal primers (Figure 1B) yielded an approximately 2047 bp fragment (including the vector sequence), with the observed band located near 2000 bp, matching the predicted size. Recombinant *E. coli* DE3 was successfully constructed following PCR identification and sequencing verification.

SDS-PAGE analysis (Figure 1C) demonstrated that the DDE protein (theoretical molecular weight: 63.51 kDa) was predominantly expressed in the soluble fraction of *E. coli* lysate. After purification using His-tag magnetic beads, a single specific band was obtained (Figure 1D). The purified DDE protein was concentrated via ultrafiltration, with its concentration determined as 408.04 μg/mL using the BCA assay.

### 2.2. Functional Validation of the DON-Degrading Enzyme DDE

**Figure 2:**
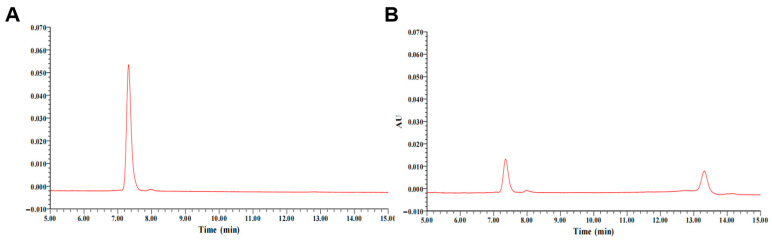
HPLC analysis of DON degradation by purified DDE. (**A**) Chromatogram of DON standard. (**B**) Chromatogram of reaction mixture after 12 h incubation (37 °C, pH 7.0) with DDE and PQQ. *Y*-axis: Absorbance Units (AUs) at 218 nm. Peaks: DON (RT = 7.36 min, 75.24 ± 3.12% reduction vs. control); 3-keto-DON (RT = 13.34 min, newly formed). All experiments were performed in triplicate (*n* = 3); chromatograms shown are representative.

As illustrated in Figure 2, co-incubation of the purified DDE with DON in the presence of PQQ resulted in a 75.24% reduction in the characteristic DON peak (retention time: 7.36 min) after a 12 h reaction. Concurrently, a novel chromatographic signal emerged at 13.34 min, corresponding to the enzymatic transformation product. These results unambiguously demonstrate the DON-degrading capacity of the purified DDE.

### 2.3. Analysis of the Enzymatic Properties of the DON-Degrading Enzyme (DDE)

**Figure 3:**
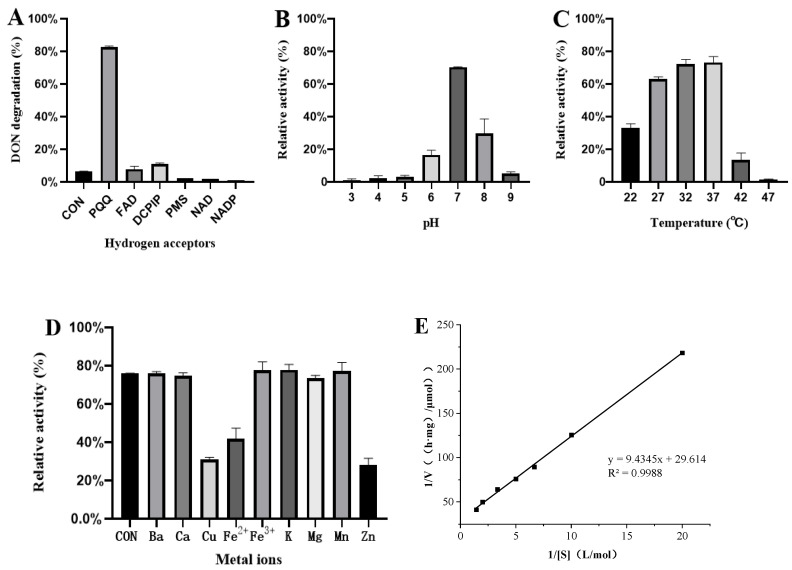
Enzymatic properties of DDE. (**A**) Cofactor specificity (12 h, 37 °C, pH 7.0). (**B**) Optimum pH (3.0–9.0). (**C**) Optimum temperature (22–42 °C). (**D**) Metal ion effects (10 mM). (**E**) Lineweaver–Burk plot. *Y*-axis: (**A**) DON degradation (%); (**B**–**D**) relative activity (%); (**E**) 1/v (min/μmol). Data are means ± SD (*n* = 3).

The hydrogen receptor specificity of DDE is shown in Figure 3A. In the absence of a hydrogen receptor, 6.37% of DON was degraded after 12 h (rate: 0.53%/h). With PQQ as the hydrogen receptor, 82.51% of DON was degraded after 12 h (rate: 6.88%/h). Both DepA in *Devosia* 17-2-E-8 and QDDH in *Devosia* D6-9 are PQQ-dependent dehydrogenases that can convert DON into 3-keto-DON, and the similarity between them is 100% [29,30,32]. By comparing the genomics of *Sphingomonas* sp. S3-4 and *Devosia* sp. 17-2-E-8, the gene of aldehyde ketone reductase AKR18A1 was discovered. With the participation of NADP^+^, AKR18A1 can oxidize DON to 3-keto-DON [33]. In addition, a dehydrogenase DDH with DON degradation ability was identified by comparative genomic analysis of the halotolerant marine bacteria ANSP101, *Devosia* 17-2-E-8, and *Devosia* IFO13580. DDH has 57.55% sequence identity with DepA and QDDH. TDDH, a mutant of DDH, degrades DON into 3-keto-DON with the participation of PMS, DCPIP, and PQQ, and the degradation ability of TDDH is stronger [31]. In this study, the sequence similarity between DDE and DepA was 98.32%. Similar to previous studies, DDE is a PQQ-dependent dehydrogenase.

As shown in Figure 3B, the degradation rate of DDE toward DON peaked at pH 7.0 within the tested pH range of 3.0–9.0. DDE’s enzymatic activity increased progressively with pH, reaching a maximum activity at pH 7.0. Figure 3C presents the optimal temperature profile of DDE, showing that it exhibited maximum activity at 37 °C (tested range: 22–42 °C), with activity increasing steadily up to this temperature. Figure 3D shows the effect of various metal ions on DDE activity. Under optimal conditions (pH 7.0, 37 °C), a 10 mM concentration of Cu^2+^, Fe^2+^, and Zn^2+^ significantly inhibited enzymatic activity, while other tested metal ions had no observable effect. Kinetic analysis using a Lineweaver–Burk plot (Figure 3E) yielded the equation y = 9.4345x + 29.614 (R^2^ = 0.9988), corresponding to a Michaelis constant (Km) of 0.32 mM and maximum velocity (Vmax) of 563.3 nmol/(min·mg). Previous studies reported that TDDH (requiring PQQ as a cofactor) displayed optimal DON degradation at 35 °C and pH 8.0, with kinetic parameters of Vmax = 429.7 nmol/(min·mg) and Km = 0.54 mM [31]. In contrast, the purified DDE in our study showed optimal activity at 37 °C and pH 7.0, and was inhibited by Cu^2+^, Fe^2+^, and Zn^2+^.

### 2.4. Detection of DDE-Catalyzed Metabolic Products

**Figure 4:**
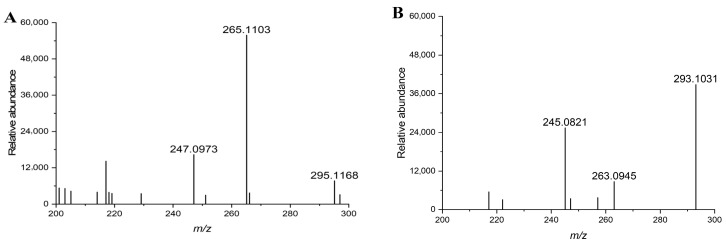
MS/MS spectra of DON and its degradation product. (**A**) DON standard. *m*/*z*: 295.1168 ([M-H]^−^), 265.1103, 247.0973. (**B**) 3-keto-DON (DDE-treated). *m*/*z*: 293.1031 ([M-H]^−^), 263.0945, 245.0821. *Y*-axis: Relative abundance.

The secondary mass spectra of DON and its degradation products are shown in Figure 4. The molecular weight of DON is 296.1254. In negative ion mode, the corresponding molecular ions are 247.0973 ([M-CH_2_O-H_2_O-H]^−^), 265.1103 ([M-CH_2_O-H]^−^), and 295.1168 ([M-H]^−^) (Figure 4A). After treatment with DDE, molecular ions corresponding to the degradation products of DON were observed at 245.0821 ([M-CH_2_O-H_2_O-H]^−^), 263.0945 ([M-CH_2_O-H]^−^), and 293.1031 ([M-H]^−^) (Figure 4B). Previous studies have identified 3-keto-DON as the product of DON degradation by AKR18A1, DepA, QDDH, and TDDH. In this study, LC-MS was used to analyze the degradation products of DDE. The secondary mass spectrometry results indicated that the molecular weight of the degradation products differed from that of DON by 2 units, and the characteristic fragment ions of the degradation products were consistent with those previously reported [31,34]. Therefore, the product of DON degradation by DDE in this experiment was identified as 3-keto-DON. Although LC-MS analysis provided strong evidence supporting the formation of 3-keto-DON, we acknowledge that LC-MS alone cannot completely confirm the structural identity of the degradation product. Future studies employing high-resolution mass spectrometry (HRMS) or nuclear magnetic resonance (NMR) will be necessary to further validate the molecular structure and confirm the degradation pathway.

### 2.5. Effects of DON-Degrading Enzymes on Serum Biochemical Parameters, Liver Function, and Intestinal Health in DON-Exposed Mice

Exposure to DON in animals not only causes acute toxicity, but may also lead to immunosuppression, hepatic injury, and gastrointestinal dysfunction. The DON-degrading enzyme (DDE) studied in this experiment showed an effective degradation capacity in vitro. At present, there are few reports on the effect of applying DON-degrading enzymes in vivo. Therefore, this study systematically explores the effects of applying DDEs on DON-exposed mice, including effects on serum physiological and biochemical parameters, liver function, and intestinal health. No adverse effects attributable to DDE were observed.

#### 2.5.1. Effects of DDE on Organ Indices in DON-Exposed Mice

Organ indices are critical biomarkers for assessing toxin-induced physiological alterations in experimental animals. As the primary metabolic and detoxification organ, the liver is particularly vulnerable. DON exposure (T1) significantly increased the hepatic index compared to the control group (46.84 ± 2.81 vs. 41.77 ± 2.27, *p* < 0.05; Table 1), indicating hepatomegaly. Notably, co-administration of DDE and PQQ (T4) fully normalized the hepatic index to control levels (43.49 ± 2.00, *p* > 0.05 vs. CON; *p* < 0.05 vs. T1). In contrast, DDE alone (T2) and PQQ alone (T3) showed only a trend toward recovery, without statistical significance versus T1. These findings suggest synergistic hepatoprotection by the DDE–PQQ combination. No significant changes were observed in cardiac, renal, or spleen indices across groups. The observed hepatomegaly in DON-exposed mice aligns with prior reports of mycotoxin-induced visceromegaly in rodents [35].

#### 2.5.2. Effects of DDE on Physiological and Biochemical Indicators in DON-Exposed Mice

Physiological and biochemical serum indices are important for assessing animal health. They reflect metabolic status, nutritional balance, and overall health. DON is mainly absorbed through the gastrointestinal tract, metabolized in the liver, and excreted via urine. In the bloodstream, DON causes organ damage by weakening the antioxidant defense system and generating free radicals, leading to oxidative stress [36]. Superoxide dismutase (SOD), glutathione peroxidase (GSH-PX), and catalase (CAT) are key antioxidant enzymes that neutralize free radicals; higher free radical levels can boost production of these enzymes. Malondialdehyde (MDA), an end product of lipid peroxidation, is a key marker for evaluating antioxidant capacity [37]. As shown in Table 2, DON-exposed mice (T1 group) had lower GSH-Px, SOD, and CAT activities (*p* < 0.05) and higher MDA levels (*p* < 0.05) in serum and liver versus the control group (CON). These results show that DON disrupts the antioxidant system and causes oxidative stress. Similar findings were observed in weaned piglets fed *Fusarium* toxin-contaminated diets, with lower SOD and GSH-Px activities and higher MDA levels in the liver and spleen [38]. In the T4 group, who were given DON, DDE, and PQQ, enzyme activities were significantly restored compared to T1 (*p* < 0.05). This suggests DDE and PQQ help reduce DON-induced oxidative stress and liver injury.

Lipid metabolism plays a crucial role in regulating blood lipid balance, cholesterol homeostasis, and lipoprotein transport. Total cholesterol (TC) represents the sum of cholesterol across all blood lipoproteins. Reduced TC levels may impair corticosteroid synthesis and consequently diminish systemic stress responses and immune function. High-density lipoprotein cholesterol (HDL-C) and low-density lipoprotein cholesterol (LDL-C) facilitate cholesterol and phospholipid transport. Decreased HDL-C levels can increase blood viscosity and promote atherosclerosis, potentially compromising liver function, while elevated LDL-C levels similarly contribute to atherosclerosis. Triglycerides (TGs), as the body’s primary energy source, may induce blood hyperviscosity, hepatic injury, and renal dysfunction at elevated levels, and are associated with decreased HDL-C and increased LDL-C levels. As shown in Table 3, compared with the CON group, the T1 group exhibited significantly lower TC and HDL-C levels (*p* < 0.05) and higher TG and LDL-C levels (*p* < 0.05), indicating DON-induced lipid metabolism dysfunction. These results align with previous reports [39,40]. PQQ alone (T3) normalized LDL-C, whereas the combination of DDE and PQQ (T4) restored TG and LDL-C to control levels (*p* > 0.05 vs. CON). No significant differences were observed between T3 and T4 for these parameters, suggesting that PQQ is the primary driver of lipid recovery under the tested conditions, while DDE may contribute subtly or synergistically when combined with PQQ.

Serum transaminases (aspartate aminotransferase, AST; alanine aminotransferase, ALT), alkaline phosphatase (AKP), and albumin (ALB) are key indicators of animal health. The levels of AST and ALT in serum can be used to assess liver damage, as these enzymes are normally found in the cytoplasm of intact liver cells and are released into the bloodstream when the liver is damaged [41]. The levels of ALT, AST, and AKP in the liver can serve as primary indicators of liver diseases, and changes in these levels reflect the state of liver function and the extent of liver cell damage [42]. Elevated creatinine (CRE) levels typically indicate poor glomerular filtration, suggesting kidney damage or reduced function. Blood urea nitrogen (BUN) is a major product following protein breakdown, and lower concentrations usually imply higher protein metabolic efficiency and utilization [43]. As shown in Table 4, compared with the CON group, the T1 group exhibited significantly higher activities of ALT, AST, and AKP in serum and liver, as well as significantly higher levels of CRE and BUN in serum (*p* < 0.05) and significantly lower levels of ALB (*p* < 0.05). Similar findings have been reported in previous studies [44,45,46], indicating that DON causes liver damage. In contrast, the T4 group, which received DDE, showed significantly lower activities of ALT, AST, and AKP in serum and the liver (*p* < 0.05) and significantly lower levels of CRE and BUN in serum (*p* < 0.05), with ALB levels returning to normal (*p* > 0.05). These results suggest that DDE helps to reduce organ damage caused by DON by breaking it down before it is absorbed into the gastrointestinal tract, thereby reducing its harmful effects. From a practical perspective, the presence of PQQ may be essential for achieving optimal DON detoxification in vivo. Therefore, co-supplementation of DDE with PQQ, or the use of microorganisms capable of naturally synthesizing PQQ, could be considered in future feed additive formulations.

Although the DON concentrations in liver and kidney tissues were determined using immunoaffinity column clean-up followed by HPLC [47], residual DON levels in the T4 group (DON + DDE + PQQ) were below the detection limit. This finding supports the occurrence of highly efficient in vivo detoxification, although formal statistical analysis could not be performed due to non-quantifiable levels.

#### 2.5.3. Effects of DDE on Intestinal and Liver Morphology in DON-Exposed Mic

**Figure 5:**
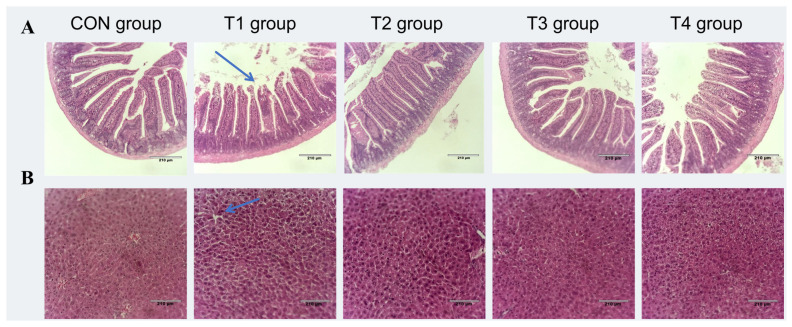
Effects of DDE on DON-induced morphological changes in the jejunum and liver of mice (H&E staining, ×100): (**A**) Jejunal sections. The blue arrow indicates reduced villus height. (**B**) Liver sections. The blue arrow indicates cytoplasmic vacuolation.

The intestine is the main organ for digesting and absorbing nutrients in animals. When animals eat DON-contaminated feed, intestinal epithelial cells are directly exposed to toxins. They are the first to be damaged. Intestinal villi help absorb nutrients, with taller villi and shallower crypts leading to better nutrient absorption, and the villus height-to-crypt depth ratio (V/C ratio) can indicate gut health [48]. As Figure 5A shows, control (CON) mice had normal jejunal villi, while T1 mice had deeper crypts, shorter villi, and damaged intestinal mucosa. Compared to T1, the T2, T3, and T4 groups exhibited longer villi, shallower crypts, and reduced mucosal damage. Table 5 shows the jejunal villus measurements. T1 has shorter villi and lower V/C than CON (*p* < 0.05), while T4 has longer villi and higher V/C than T1 (*p* < 0.05). The crypt depth does not differ between groups (*p* > 0.05). These results show that DON damages mouse jejunal villi and lowers the V/C ratio by entering intestinal epithelial cells, damaging the intestinal wall, and preventing villi growth. This harms the gut’s structure and function, weakening its physical barrier [49]. Our findings are similar to those of past studies, where piglets fed 2.65 mg/kg DON had shorter villi, a lower V/C ratio, and deeper crypts [50]. Broilers fed DON also had shorter villi [51]. This experiment shows that DDE + PQQ (T4) most effectively mitigates DON-induced reductions in the villus height and V/C ratio, thereby preserving the intestinal physical barrier.

The liver is the primary organ responsible for metabolizing DON and is therefore highly susceptible to its toxic effects. Specifically, this organ preferentially accumulates substantial quantities of DON and its metabolites, ultimately inducing tissue damage. Previous studies have shown that rats exposed to 0, 1, 5, and 10 mg/kg of DON exhibited liver tumors and necrosis [52]. As shown in Figure 5B, compared to the CON group, mice in the T1 group showed varying degrees of cytoplasmic vacuolization in hepatocytes, evidenced by the absence of cytoplasmic staining. In the T2 and T3 groups, the degree of cytoplasmic vacuolization was reduced, and the number of erythrocytes in the hepatic sinusoids decreased. In the T4 group, the hepatocytes showed a slight increase in lymphocytes and an increase in erythrocytes in the hepatic sinusoids. Pathological changes in the liver were significantly reduced, indicating that DDE can significantly alleviate the liver damage caused by DON in mice.

#### 2.5.4. Effects of DDE on mRNA Expression of Genes Associated with Intestinal Inflammation, Barrier Function, and Apoptosis in DON-Exposed Mice

**Figure 6:**
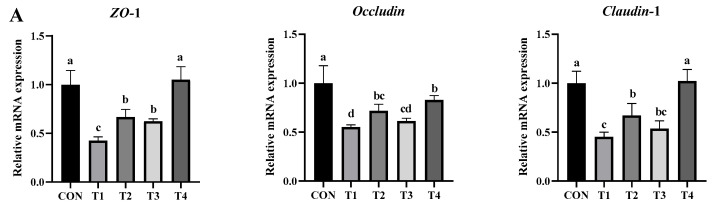

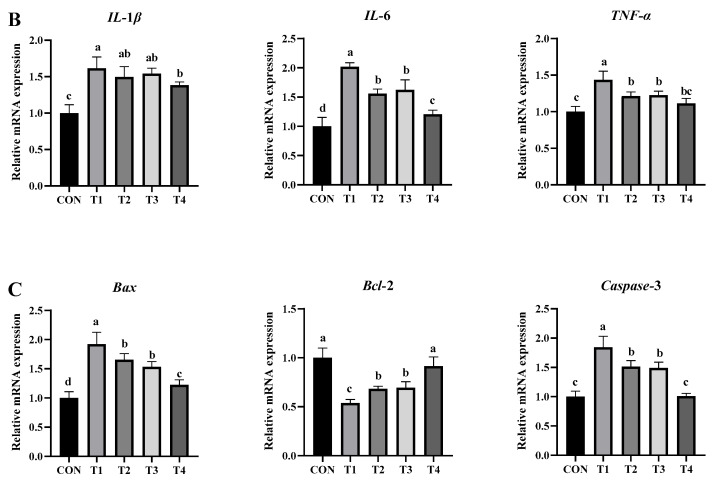
Effects of DDE on the relative mRNA expression of jejunal genes in DON-exposed mice: (**A**) barrier-related genes; (**B**) inflammation-related genes; (**C**) apoptosis-related genes. Note: Identical letters or no letters indicate no significant difference (*p* > 0.05), whereas different lowercase letters denote significant differences (*p* < 0.05). Data are means ± SD (*n* = 6 per group).

The intestinal mechanical barrier is mainly made of tight junction complexes and the intestinal epithelium. Claudins, Occludin, and ZO are key proteins in tight junction complexes; if these proteins are less expressed, gut permeability increases, harming the intestinal barrier’s integrity. To see how DDE affects the intestinal barrier in DON-exposed mice, we measured mRNA for tight junction genes (*ZO-1*, *Occludin*, *Claudin-1*) in the jejunum. Figure 6A shows that the *ZO-1*, *Occludin*, and *Claudin-1* levels in the DON group (T1) were significantly lower than those in the control (CON) (*p* < 0.05), indicating that DON badly damages the intestinal barrier. Past studies corroborate these results. For example, long-term DON exposure was found to lower Occludin and raise pro-inflammatory cytokines (TNF-α, IL-1β, IL-6) in piglets [53], while another study found that DON breaks the mouse jejunal barrier by lowering *ZO-1* and *Claudin-1* [6]. Compared to T1, T2 and T4 had higher *ZO-1*, *Occludin*, and *Claudin-1* levels (*p* < 0.05), while T3 only had higher *ZO-1* levels (*p* < 0.05). These results show that DDE, especially with PQQ, helps to reduce DON damage. It may maintain the intestinal barrier by increasing tight junction proteins.

The intestine is the body’s biggest immune organ, and its immune barrier is made of various immune cells [54]. Inflammatory factors like IL-1β, IL-6, and TNF-α are key for waking up immune cells and starting immune responses. When these factors are overexpressed, they bring about chronic inflammation and tissue damage. IL-6 not only activates immune cells but also delays the normal phagocytic function of Phagocytes, thereby exacerbating inflammatory responses [55]. As shown in Figure 6B, compared to the control group (CON), the DON-exposed group (T1) showed significantly increased expression of *IL-1β*, *IL-6*, and *TNF-α* (*p* < 0.05), indicating that DON exposure induces inflammatory responses in the mouse jejunum [53,56]. Compared to the T1 group, the T2 and T3 groups exhibited significantly reduced expressions of *IL-6* and *TNF-α* (*p* < 0.05), with T4 showing the greatest reduction in *IL-1β*, *IL-6*, and *TNF-α*, returning to near-normal levels. These results indicate that both DDE and PQQ individually provide partial anti-inflammatory effects, while their combination leads to the most pronounced suppression of DON-induced intestinal inflammation. Notably, the T2 group (DON + DDE without exogenous PQQ) still exhibited partial protection, as reflected by lower *IL-6* and *TNF-α* expression compared with the T1 group. This residual effect may be attributed to trace amounts of endogenous PQQ in the host, sustaining the limited DDE activity, or to potential non-enzymatic adsorption or detoxification properties of DDE itself. Collectively, these findings suggest that co-administration of DDE with PQQ provides the most effective protection and offers valuable guidance for practical application of DON-degrading enzymes.

Intestinal epithelial cell homeostasis is fundamental to the barrier function of the gut. DON-induced oxidative stress can lead to epithelial cell damage and subsequently trigger apoptosis. Bcl-2, an anti-apoptotic protein located on the outer mitochondrial membrane, and Bax, a pro-apoptotic protein in the cytoplasm, are critical regulators of apoptosis, with their relative ratio being a key determinant of the apoptotic process. Caspase-3, a major implementer of apoptosis, is activated to cleave multiple cellular substrates, ultimately resulting in programmed cell death [57]. Figure 6C illustrates the effects of DDE on the relative mRNA expression of apoptosis-related genes in the jejunum of DON-exposed mice. Compared with the control (CON) group, the T1 group showed a significant upregulation of *Bax* and *Caspase-3* (*p* < 0.05), accompanied by a significant downregulation of *Bcl-2* (*p* < 0.05). Similar results have been reported previously [58], indicating that DON exposure promotes the expression of apoptosis-related genes in the mouse jejunum. Moreover, PQQ has been shown to alleviate inflammation, attenuate oxidative stress, and exert anti-apoptotic effects [59]. This may explain why the T3 and T4 groups exhibited significantly lower expression of *Bax* and *Caspase-3* (*p* < 0.05) and higher expression of *Bcl-2* (*p* < 0.05) relative to the T1 group. Notably, no significant differences in *Bcl-2* or *Caspase-3* expression were detected between the T4 and CON groups (*p* > 0.05), suggesting that DDE, together with PQQ, effectively mitigates DON-induced apoptosis and thereby preserves the intestinal barrier function.

#### 2.5.5. Effects of DDE on the Gut Microbiota of DON-Induced Mice

A healthy gut microbiota is essential for maintaining the intestinal mucosal barrier, promoting nutrient absorption and metabolism, regulating the immune system, and inhibiting the growth of pathogenic microorganisms [60]. Mycotoxins may suppress the growth of certain gut microbes, disrupt their normal metabolic activities, and reduce their relative abundance. Prolonged exposure to mycotoxins can lead to a decrease in gut microbial diversity [61].

**Figure 7:**
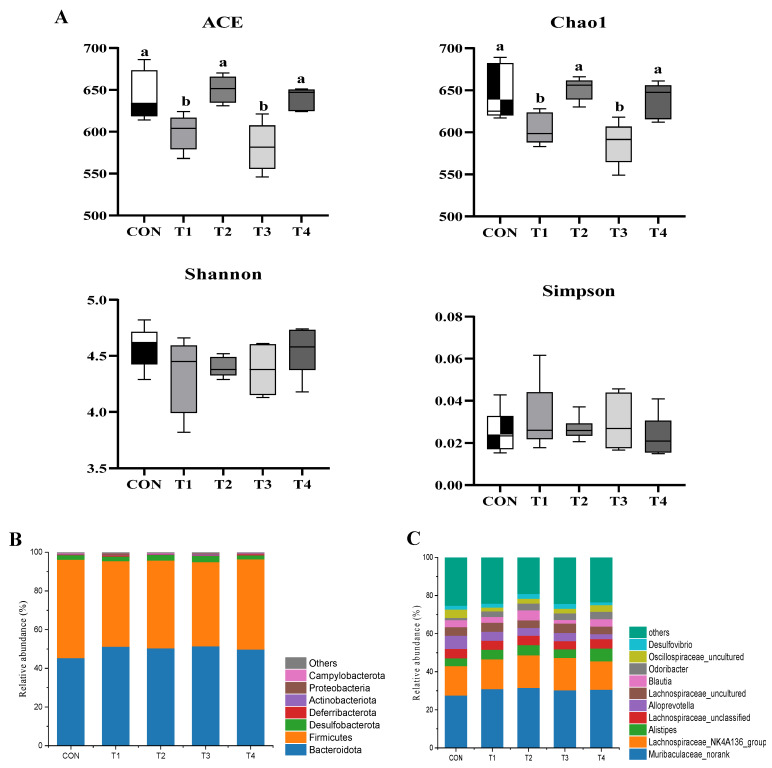
Effects of DDE on gut microbiota diversity in DON-exposed mice (*n* = 6). (**A**) Alpha diversity indices (ACE, Chao1, Shannon, Simpson) Note: Identical letters or no letters indicate no significant difference (*p* > 0.05), whereas different lowercase letters denote significant differences (*p* < 0.05); (**B**) microbial composition at the phylum level; (**C**) microbial composition at the genus level.

Alpha diversity is an important indicator for evaluating species diversity within a specific ecosystem, and indicates how even and rich species are. A higher alpha diversity usually means a more stable microbial community. This helps keep pathogens out and boosts the immune function [62]. The ACE and Chao1 indices indicate species richness, while the Shannon and Simpson indices show richness and evenness. Figure 7A shows that the CON, T2, and T4 groups had higher ACE and Chao1 indices than T1 and T3 (*p* < 0.05), while the Shannon and Simpson indices showed no significant difference between groups (*p* > 0.05). DON exposure reduced ACE and Chao1 indices in the mouse intestines, indicating that DON reduced gut microbe diversity. Past studies [63,64] reported similar findings, observing that DON reduced gut microbiota diversity in rabbits and piglets. The addition of DDE somewhat reverted the changes in ACE and Chao1, suggesting that DDE can counter DON’s negative effects on gut microbe diversity.

The gut microbiota plays a key role in digestion, absorption, metabolism, and immune defense in animals. A balanced microbial environment helps with nutrient absorption, can synthesize essential nutrients, and protects the host from pathogens. Gut microbes release different enzymes to break down food and also make metabolites such as short-chain fatty acids, i.e., butyric and propionic acid. These give energy to the host and reduce inflammation [65]. For example, butyric acid from Bacteroidetes helps digestion, lowers inflammation, and helps with obesity [66]. Firmicutes, meanwhile, play a part in energy metabolism and maintaining the gut barrier [67]. As shown in Figure 7B, the main gut microbes in mice at the phylum level are Bacteroidetes, Firmicutes, Desulfobacterota, Deferribacteres, Actinobacteria, Proteobacteria, and Campylobacterota. Together, they make up over 99% of the microbiota, with Firmicutes and Bacteroidetes being the most common. This corroborates the findings of Zhang et al. [68]. In addition, compared to CON, T1 had less Firmicutes and more Bacteroidetes (*p* < 0.05), and other studies saw similar results [69,70]. Firmicutes are important for energy metabolism and gut health [67]. In this study, DON disrupted the gut microbiome by reducing Firmicutes and increasing Bacteroidetes. This might cause intestinal inflammation and metabolic problems. While DDE supplementation seemed to boost Firmicutes, the effect was not significant. This could be due to DDE’s purity or dosage.

As shown in Figure 7C, the gut microbiota of mice at the genus level is primarily composed of ten taxa: Muribaculaceae_norank, Lachnospiraceae NK4A136 group, Alistipes, Lachnospiraceae unclassified, Alloprevotella, Lachnospiraceae_uncultured, Blautia, Odoribacter, Oscillospiraceae_uncultured, and Desulfovibrio. The relative abundance of Odoribacter and Oscillospiraceae_uncultured was significantly higher in the CON and T4 groups compared to the T1, T2, and T3 groups (*p* < 0.05). Notably, Muribaculaceae, Alistipes, and Alloprevotella, which were highly abundant, can produce short-chain fatty acids (SCFAs) through metabolism. These metabolites are crucial for maintaining the intestinal barrier function, suppressing inflammation, and regulating host metabolism. For example, Muribaculaceae, a dominant genus in healthy individuals, directly improves the gut environment through its fermentation products [71]. Alistipes can reduce blood lipid and glucose levels, but its role in obesity remains controversial, with some studies suggesting it may promote it [72,73,74]. Additionally, Odoribacter, a typical SCFA-producing genus, exhibits a negative correlation with metabolic diseases such as non-alcoholic fatty liver disease and inflammatory bowel disease. It can also alleviate obesity-related inflammation by consuming succinate and improving glucose tolerance [75,76]. In this study, DON exposure significantly reduced the abundance of Odoribacter, while DDE intervention significantly restored its abundance (*p* < 0.05), indicating that DDE may alleviate DON-induced gut microbiota dysbiosis by promoting the proliferation of specific beneficial bacteria. Although the increase in Firmicutes abundance with DDE supplementation did not reach statistical significance, the restoration of Odoribacter suggests that DDE has potential applications in modulating gut microbiota balance.

## 3. Conclusions

In this study, the DON-degrading enzyme (DDE) from *Devosia* sp. JA3 was successfully cloned and expressed. It was found that DDE led to an 82.51% degradation in DON within 12 h in the presence of PQQ, with 3-keto-DON as the degradation product. The optimal pH for DDE activity was 7.0, and the optimal temperature was 37 °C. Moreover, Cu^2+^, Fe^2+^, and Zn^2+^ were shown to inhibit the degradation of DON by DDE, and its kinetic parameters were superior to those of most DON-degrading enzymes. Additionally, DDE significantly alleviated DON-induced oxidative damage, liver dysfunction, and intestinal barrier impairment in mice, while modulating the gut microbiota and improving intestinal health. The results indicate that DDE possesses an efficient DON-degrading ability and good food safety, providing comprehensive scientific evidence for the development and application of DON-degrading enzymes, with significant theoretical and practical implications. Given the excellent health recovery observed in DON + DDE-treated mice, DDE is functionally safe for use; however, future studies should incorporate a DDE-only control group to provide definitive biosafety confirmation.

## 4. Materials and Methods

### 4.1. DON-Degrading Strains and Vectors

The *Devosia* sp. JA3 strain was provided by Henan Yiwan Zhongyuan Biotechnology Co., Ltd., Zhengzhou, China. *Escherichia coli* DH5α and *Escherichia coli* BL21 (DE3) were purchased from Sangon Biotech (Shanghai) Co., Ltd., Shanghai, China. The pET-31b vector was obtained from Novagen (Madison, WI, USA). Isopropyl β-D-1-thiogalactopyranoside (IPTG) and ampicillin (Amp) were purchased from Shanghai Macklin Biochemical Technology Co., Ltd., Shanghai, China.

### 4.2. Heterologous Expression of DON-Degrading Enzyme DDE

#### 4.2.1. Cloning of the DDE Gene and Construction of the Recombinant Vector

The genomic DNA of *Devosia* sp. JA3 was extracted and subjected to whole-genome sequencing. Based on the reported DON-degrading enzyme DepA (KFL25551.1), degenerate primers for the DDE gene were designed. The upstream primer sequence was TGGTGGTGGTGGTGCTATGGGGCTTGCCCTTTCGACT, and the downstream primer sequence was TTTAACTTTAAGAAGGAGATATAC AGCTTGGCTTCGGGCA. *Nde I* and *Xho I* cleavage sites were added to both ends of the primers, as were homology arms. PCR amplification was performed using a thermal cycling program and PCR products were separated by 1% agarose gel electrophoresis. Subsequently, DNA was recovered from the gel, and the plasmid pET-31b was then double-digested with *Nde I* and *Xho I*. The digestion products were separated and purified using 1% agarose gel electrophoresis, followed by gel DNA recovery. The recombinant plasmid pET-31b-DDE was constructed by ligating the linearized plasmid pET-31b with the target gene fragment using the ClonExpress Ultra One-Step Cloning Kit. The recombinant plasmid was then transformed into *Escherichia coli* DH5α using the heat shock method [77]. Single colonies were picked and inoculated into LB-Amp liquid medium, followed by incubation at 37 °C and 200 rpm for 12 h for colony PCR screening of positive clones. The universal primers T7 (TAATACGACTCACTA TAGGG) and T7-ter (GCTAGTTATTGCTCAGCGG) were used for colony PCR. The PCR products were analyzed by 1% agarose gel electrophoresis, and those matching the size of the target gene were sent for sequencing. The correctly sequenced recombinant plasmid pET-31b-DDE was then transformed into *E. coli* BL21 (DE3) using the heat shock method.

#### 4.2.2. Expression and Purification of the Degrading Enzyme (DDE)

The DE3 strain, preserved in glycerol, was inoculated into LB-Amp liquid medium for activation. Subsequently, the activated bacterial culture was transferred at a 5% inoculation ratio into fresh LB-Amp medium and cultured at 37 °C and 200 rpm for 2–3 h until the OD_600_ value reached 0.6–0.8. IPTG was then added to the medium at a final concentration of 0.4 mmol/L for induction. Following induction, bacterial cells were concentrated using centrifugation (4 °C, 10,000 rpm, 30 min). Finally, the cells were resuspended in binding buffer and subjected to ultrasonic disruption on ice. The resulting supernatant after disruption was collected as the crude DDE solution.

Next, protein purification was performed using magnetic beads. Gradient elution was carried out using different ratios of elution and binding buffers (0%, 5%, 10%, 20%, 50%, and 100%). The eluted fractions underwent analysis via sodium dodecyl sulfate-polyacrylamide gel electrophoresis (SDS-PAGE). Specifically, a 12% separation gel and 5% stacking gel were prepared using an SDS-PAGE gel kit for electrophoresis. Following electrophoresis, the gel was subjected to staining with Coomassie Brilliant Blue R-250, followed by destaining, with multiple changes of destaining solution until the protein bands were clearly visible.

#### 4.2.3. Validation of DDE-Degrading Enzyme Activity

Reactions were conducted in a 500 μL system comprising 100 μL of purified degrading enzyme, 0.1 mg PQQ, and 5 μg DON, with a control reaction omitting DDE. The mixture was incubated at pH 7.0 and 37 °C for 12 h. The residual DON content was quantified by HPLC under the following conditions: column, Diamonsil^®^ C18 reversed-phase column; injection volume, 30 μL; column temperature, 35 °C, flow rate, 1 mL/min; and detection wavelength, 218 nm. The gradient elution program featured a mobile phase of water:acetonitrile (88:12, *v*/*v*) as follows: 0–15 min, 12–33% acetonitrile; 15–16 min, 33–90% acetonitrile; 16–18 min, 90% acetonitrile; 18–19 min, 90–12% acetonitrile; followed by a 4 min hold at 12%.

#### 4.2.4. Determination of DDE Degradation Enzyme Concentration

The DDE was concentrated using a 10 kDa ultrafiltration tube via centrifugation at 4 °C, 4500 rpm for 60 min, followed by three washes with phosphate-buffered saline (PBS). The concentration of DDE was determined using the bicinchoninic acid assay (BCA) method. The protein concentration exhibited excellent linearity within the range of 0–0.5 mg/mL, with a correlation coefficient of R^2^ = 0.9986 and a regression equation of y = 0.9830x + 0.1579. For sample measurement, 20 μL of purified degrading enzyme was mixed with 200 μL of BCA working solution, and the absorbance at 562 nm was measured. The protein concentration of the purified sample was calculated by interpolation from the standard curve.

#### 4.2.5. Analysis of the Properties of DDE

The hydrogen acceptor specificity of DDE was investigated by mixing a 20 μL aliquot of DDE with 50 μg/mL DON, supplemented with 500 μM of individual hydrogen acceptors (PQQ, PMS, DCPIP, NAD, NADP, or FAD). The reactions were conducted in a pH 7.0 buffer at 37 °C for 12 h, with parallel controls that omitted the hydrogen acceptors.

To determine the optimum pH for DDE activity, reaction buffers were prepared at pH 3 (citric acid and sodium citrate), pH 4–8 (potassium dihydrogen phosphate and dipotassium hydrogen phosphate), and pH 9 (glycine and sodium hydroxide). DDE and DON were added to each buffer, and the reactions were carried out at 37 °C for 12 h.

The optimal temperature for DDE activity was examined by incubating DDE and DON in phosphate buffer (50 mM, pH 7.0) at various temperatures: 22, 27, 32, 37, and 42 °C, for 12 h.

To study the effects of metal ions on DDE activity, DDE and DON were mixed in phosphate buffer (pH 7.0), and solutions of BaCl_2_, CaCl_2_, CuSO_4_, FeSO_4_, Fe_2_(SO_4_)_3_, KCl, MgCl_2_, MnSO_4_, and ZnCl_2_ were added to achieve a final concentration of 10 mM. A control reaction without any added metal ions was also conducted. All mixtures were then incubated for 12 h.

Finally, the Km and maximum reaction rate Vmax of the DON-degrading enzyme DDE were determined by mixing DDE with DON at six different concentrations (50, 100, 150, 200, 250, and 500 µM) in 500 µL of phosphate buffer (50 mM, pH 7.0). The reactions were performed at 37 °C for 12 h to calculate the Km and Vmax values.

#### 4.2.6. LC-MS Analysis of DDE Degradation Products

An equimolar solution (200 µg/mL each) of DDE and DON was reacted in 50 mM phosphate buffer (pH 7.0) at 37 °C for 24 h. The mixture underwent triple extraction with equal volumes of ethyl acetate. The pooled organic phases were rotary evaporated to dryness at 40 °C, reconstituted in HPLC-grade methanol, and filtered through a 0.22 µm PTFE membrane before LC-MS analysis.

Liquid Chromatography–Mass Spectrometry (LC–MS) Conditions: Chromatographic separation was carried out on a Dionex Ultimate 3000 UHPLC system (Thermo Scientific, Waltham, MA, USA) equipped with an Atlantis T3 column (100 mm × 3.0 mm, 3.0 μm). The binary mobile phase consisted of (A) 5 mM ammonium formate with 0.1% formic acid in water and (B) acetonitrile. The gradient elution program was as follows: 0–1 min, 90% A; 1–6 min, 90–10% A; 6–7 min, 10% A; 7–8 min, 10–90% A. The flow rate was 0.4 mL/min, the column temperature was maintained at 30 °C, and the injection volume was 10 μL.

Mass spectrometric detection was performed using a Q Exactive mass spectrometer (Thermo Scientific, Waltham, MA, USA) equipped with a heated electrospray ionization (HESI^+^) source. The spray voltage was 3.0 kV, the capillary temperature was 320 °C, and the sheath and auxiliary gas flow rates were 35 and 10 arbitrary units, respectively. The S-lens RF level was set to 50. Full-scan data were acquired over an *m*/*z* range of 80–1200 at a resolution of 70,000 (*m*/*z* 200). Data acquisition and processing were performed using Xcalibur software (Thermo Fisher Scientific, Waltham, MA, USA; version 4.6).

### 4.3. Effects of DON-Degrading Enzymes on DON-Induced Hepatointestinal Injury in Mice

#### 4.3.1. Experimental Animals and Management

A total of forty male C57BL/6J mice aged 4 weeks (mean body weight 16.8 ± 1.3 g) were obtained from Liaoning Changsheng Biotechnology Co., Ltd. (license number SCXK (Liaoning) 2020-0001, Shenyang, China). All animal experiments were approved by the Animal Ethics Committee of Henan University of Technology (Approval No: IACUC-HAUT-2023-0624). Following random allocation into five experimental groups (*n* = 8 per group), all animals underwent a 7-day acclimation period under standard laboratory conditions (ambient temperature of 25 ± 1 °C, relative humidity of 50 ± 10%, and a 12 h light/dark cycle), during which individuals exhibiting abnormal behavioral patterns or health status were excluded. Throughout the study, mice were housed in appropriate cages with free access to standard laboratory chow and water. At the end of the experiment, all mice were euthanized by cervical dislocation.

#### 4.3.2. Experimental Design

Forty male C57BL/6J mice were randomly assigned to five experimental groups (*n* = 8 per group): the CON group (control, PBS, 10 mL/kg body weight (bw)), the T1 group (DON, 2 mg/kg bw/day), the T2 group (DON + DDE, 2 and 10 mg/kg bw/day), the T3 group (DON + PQQ, 2 and 6 mg/kg bw/day), and the T4 group (DON + DDE + PQQ, 2, 10, and 6 mg/kg bw/day). All components (DON, DDE, and PQQ) were administered separately by intragastric gavage without prior mixing to ensure that DDE exerted its DON-degrading activity in vivo within the gastrointestinal tract. All treatments were conducted once daily for 14 consecutive days, during which the health status and behavior of the mice were observed and recorded daily.

#### 4.3.3. Sample Collection and Processing

Mice were fasted for 12 h prior to sample collection. After weighing, blood was collected via ocular puncture. The blood samples were allowed to clot at 4 °C for 4 h and then centrifuged at 3500 rpm for 15 min. Serum was aliquoted (200 μL per tube) and stored at −80 °C. Following blood collection, mice were euthanized by cervical dislocation. The heart, liver, kidneys, and spleen were excised via laparotomy, trimmed of adipose and connective tissues, and weighed to calculate organ indices. Liver samples were fixed in 4% paraformaldehyde (PFA) for 24 h. A 0.5 cm segment of the mid-jejunum was fixed in 4% PFA, while the remaining portion was rapidly frozen in liquid nitrogen and stored at −80 °C. Cecal chyme was collected into sterile cryovials, snap-frozen in liquid nitrogen, and preserved at −80 °C until analysis.

#### 4.3.4. Measurement Parameters and Analytical Methods

Serum antioxidant indicators, including the activities of CAT, GSH-Px, and SOD, as well as the content of MDA, were measured using kits from Nanjing Jiancheng Bioengineering Institute (Nanjing, China). Serum biochemical indicators, including ALT, AST, AKP, ALB, CRE, and BUN, were measured using the corresponding kits. Serum lipid metabolism indicators, including TC, TG, HDL-C, and LDL-C, were measured using the corresponding kits. For liver antioxidant indicators, mouse livers were homogenized at 60 Hz (70 s working, 15 s resting) to prepare a 10% homogenate. The homogenate was centrifuged at 5000 rpm for 15 min at 4 °C, and the supernatant was collected and stored for further use. Protein concentration in the homogenate was determined using the BCA method, and antioxidant indicators were measured as described for serum. Liver function indicators, including ALT, AST, and AKP, were measured according to the kits’ instructions. Tissue samples fixed in 4% paraformaldehyde were dehydrated, embedded in paraffin, sectioned, stained with HE, and observed under an optical microscope for histopathological features.

Determination of organ index: Organ index = organ weight (mg)/fasting body weight (g).(1)

Determination of relative expression of mRNA related to jejunal inflammation, intestinal barrier, and apoptosis: Approximately 50 mg of jejunal tissue samples was collected, and total RNA was extracted using the Vazyme FreeZol Reagent kit. The purity and concentration of total RNA were determined using RNase-Free ddH_2_O as a blank control. The RNA samples were subjected to reverse transcription according to the relevant kit instructions. RT-qPCR was performed using the SYBR Green Premix Pro Tag HS qPCR Kit, and the relative gene expression was calculated with 2^−ΔΔCt^ math. The primers used in this assay are listed in Table 6.

Detection of gut microbiota: Samples of intestinal contents were sent to Shanghai Yuanshen Biomedical Technology Co., Ltd. (Shanghai, China) for microbial diversity analysis.

### 4.4. Data Processing and Statistical Analyses

Data processing was performed using Excel 2019, and statistical analyses were conducted using SPSS 22.0. Statistical differences among the data were determined by one-way ANOVA or independent sample t-tests. When significant differences were detected, multiple comparisons between groups were further conducted using Tukey’s test, with *p* < 0.05 indicating a significant difference. Double-reciprocal plots were created using Origin 2022 software.

## Figures and Tables

**Table 1:** Effects of DDE and PQQ on organ indices in DON-exposed mice (mean ± SD, *n* = 8).

Item	CON Group	T1 Group	T2 Group	T3 Group	T4 Group	*p*-Value
Cardiac index	6.12 ± 0.87	6.18 ± 0.62	5.70 ± 0.65	6.28 ± 1.22	5.84 ± 0.76	0.626
Hepatic index	41.77 ± 2.27 ^b^	46.84 ± 2.81 ^a^	43.86 ± 1.99 ^ab^	43.91 ± 1.89 ^ab^	43.49 ± 2.00 ^b^	0.002
Renal index	12.87 ± 2.52	13.35 ± 0.81	13.73 ± 0.56	13.85 ± 0.63	13.75 ± 0.61	0.534
Spleen index	3.01 ± 0.28	3.06 ± 0.17	3.08 ± 0.32	2.99 ± 0.24	3.12 ± 0.52	0.932

Note: Different lowercase letters within the same row indicate significant differences (*p* < 0.05). CON: control (PBS); T1: DON; T2: DON + DDE; T3: DON + PQQ; T4: DON + DDE + PQQ.

**Table 2:** Effects of DDE on DON-induced antioxidant activity in serum and liver of mice (mean ± SD, *n* = 8).

Item	CON Group	T1 Group	T2 Group	T3 Group	T4 Group	*p*-Value
Serum GSH-Px (U/mL)	1612.25 ± 57.09 ^a^	1315.19 ± 56.79 ^c^	1380.04 ± 41.55 ^c^	1485.45 ± 46.92 ^b^	1557.14 ± 56.55 ^ab^	<0.001
Liver GSH-Px (U/mg protein)	816.56 ± 26.02 ^a^	625.09 ± 41.51 ^c^	713.82 ± 20.10 ^b^	720.87 ± 19.40 ^b^	796.79 ± 39.81 ^a^	<0.001
Serum SOD (U/mL)	31.93 ± 1.64 ^a^	26.47 ± 0.62 ^c^	27.41 ± 1.16 ^c^	29.64 ± 2.17 ^b^	29.90 ± 0.88 ^ab^	<0.001
Liver SOD (U/mg protein)	9.59 ± 0.82 ^a^	7.01 ± 0.72 ^b^	7.70 ± 0.57 ^b^	7.72 ± 0.59 ^b^	9.12 ± 0.56 ^a^	<0.001
Serum CAT (U/mL)	29.80 ± 1.78 ^b^	26.47 ± 1.10 ^c^	25.67 ± 0.95 ^c^	42.12 ± 2.72 ^a^	28.43 ± 1.52 ^b^	<0.001
Liver CAT (U/mg protein)	86.94 ± 7.36 ^a^	62.86 ± 6.83 ^d^	66.45 ± 5.21 ^cd^	73.01 ± 7.61 ^bc^	81.18 ± 4.39 ^ab^	<0.001
Serum MDA (nmol/mL)	10.85 ± 0.46 ^c^	23.27 ± 2.59 ^a^	13.55 ± 1.54 ^b^	14.68 ± 0.98 ^b^	11.15 ± 0.90 ^c^	<0.001
Liver MDA (nmol/mg protein)	3.91 ± 0.55 ^b^	5.11 ± 0.95 ^a^	4.95 ± 0.72 ^ab^	5.07 ± 0.55 ^a^	4.27 ± 0.47 ^ab^	0.006

Note: Different lowercase letters within the same row indicate significant differences (*p* < 0.05). CON: control (PBS); T1: DON; T2: DON + DDE; T3: DON + PQQ; T4: DON + DDE + PQQ.

**Table 3:** Effects of DDE on serum lipid metabolism induced by DON in mice (mean ± SD, *n* = 8).

Item	CON Group	T1 Group	T2 Group	T3 Group	T4 Group	*p*-Value
TC (mmol/L)	4.10 ± 0.24 ^a^	3.50 ± 0.16 ^b^	3.52 ± 0.15 ^b^	3.78 ± 0.28 ^ab^	3.81 ± 0.23 ^ab^	<0.001
TG (mmol/L)	1.34 ± 0.13 ^b^	1.68 ± 0.12 ^a^	1.41 ± 0.12 ^b^	1.53 ± 0.11 ^ab^	1.34 ± 0.18 ^b^	<0.001
HDL-C (mmol/L)	2.61 ± 0.29 ^a^	1.62 ± 0.19 ^b^	1.75 ± 0.37 ^b^	1.91 ± 0.25 ^b^	1.97 ± 0.37 ^b^	<0.001
LDL-C (mmol/L)	3.87 ± 0.53 ^b^	4.78 ± 0.65 ^a^	4.45 ± 0.25 ^ab^	4.03 ± 0.31 ^b^	3.89 ± 0.58 ^b^	0.004

Note: Different lowercase letters within the same row indicate significant differences (*p* < 0.05). CON: control (PBS); T1: DON; T2: DON + DDE; T3: DON + PQQ; T4: DON + DDE + PQQ.

**Table 4:** The effect of DDE on serum and hepatic biochemical indices in DON-exposed mice (mean ± SD, *n* = 8).

Item	CON Group	T1 Group	T2 Group	T3 Group	T4 Group	*p*-Value
Serum ALT (U/L)	6.93 ± 1.04 ^b^	13.44 ± 1.42 ^a^	7.99 ± 0.23 ^b^	8.26 ± 0.64 ^b^	7.52 ± 1.03 ^b^	<0.001
Hepatic ALT (U/mg prot)	2.87 ± 0.07 ^d^	4.12 ± 0.37 ^a^	2.11 ± 0.24 ^b^	3.20 ± 0.60 ^bc^	2.59 ± 0.12 ^cd^	<0.001
Serum AST (U/L)	7.83 ± 0.68 ^b^	12.24 ± 0.78 ^a^	11.61 ± 0.78 ^a^	10.60 ± 1.57 ^a^	7.53 ± 2.04 ^b^	<0.001
Hepatic AST (U/mg prot)	2.57 ± 0.42 ^b^	3.55 ± 0.47 ^a^	2.99 ± 0.15 ^b^	3.09 ± 0.44 ^ab^	2.93 ± 0.12 ^b^	<0.001
Serum AKP (King units/100 mL)	9.15 ± 2.03 ^c^	18.06 ± 1.30 ^a^	13.10 ± 1.30 ^b^	10.99 ± 0.93 ^bc^	10.54 ± 1.13 ^c^	<0.001
Hepatic AKP (King units/mg prot)	7.11 ± 0.55 ^d^	14.91 ± 1.41 ^a^	13.63 ± 0.47 ^b^	13.54 ± 0.60 ^b^	10.82 ± 0.40 ^c^	<0.001
ALB (g/L)	31.33 ± 1.71 ^a^	27.86 ± 1.42 ^c^	29.08 ± 0.96 ^bc^	28.88 ± 1.49 ^bc^	30.63 ± 0.50 ^ab^	<0.001
CRE (μmol/L)	16.43 ± 0.75 ^b^	29.22 ± 3.64 ^a^	27.79 ± 2.71 ^a^	25.04 ± 3.28 ^a^	17.17 ± 1.69 ^b^	<0.001
BUN (mmol/L)	4.94 ± 0.66 ^c^	8.31 ± 0.79 ^a^	7.56 ± 1.21 ^ab^	6.27 ± 0.99 ^bc^	5.65 ± 1.21 ^c^	<0.001

Note: Different lowercase letters within the same row indicate significant differences (*p* < 0.05). CON: control (PBS); T1: DON; T2: DON + DDE; T3: DON + PQQ; T4: DON + DDE + PQQ.

**Table 5:** Effects of DDE on jejunal histomorphology in DON-exposed mice (mean ± SD, *n* = 8).

Item	CON Group	T1 Group	T2 Group	T3 Group	T4 Group	*p*-Value
Villus Height (μm)	393.40 ± 12.23 ^a^	280.39 ± 6.74 ^d^	320.54 ± 13.19 ^c^	322.60 ± 8.56 ^c^	353.74 ± 10.87 ^b^	<0.001
Crypt Depth (μm)	95.54 ± 3.08	96.15 ± 4.44	100.43 ± 2.15	97.28 ± 2.69	96.90 ± 2.70	0.095
Villus/Crypt Ratio	4.12 ± 0.12 ^a^	2.92 ± 0.12 ^d^	3.19 ± 0.12 ^c^	3.32 ± 0.14 ^c^	3.65 ± 0.16 ^b^	<0.001

Note: Different lowercase letters within the same row indicate significant differences (*p* < 0.05). CON: control (PBS); T1: DON; T2: DON + DDE; T3: DON + PQQ; T4: DON + DDE + PQQ.

**Table 6:** RT-qPCR primer sequences.

Gene	Primer Sequences (5′-3′)	Product Length (bp)	NCBI Accession Number
*β-actin*	F: ATATCGCTGCGCTGGTCG R: TTCCCACCATCACACCCTGG	129	NM_007393.5
*IL-1β*	F: AATGCCACCTTTTGACAGTGATG R: AGCTTCTCCACAGCCACAAT	189	NM_008361.4
*IL-6*	F: TAGTCCTTCCTACCCCAATTTCC R: TTGGTCCTTAGCCACTCCTTC	76	NM_031168.2
*TNF-α*	F: GACGTGGAACTGGCAGAAGAG R: TTGGTGGTTTGTGAGTGTGAG	228	NM_013693.3
*ZO-1*	F: GCCGCTAAGAGCACAGCAA R: TCCCCACTCTGAAAATGAGGA	134	NM_001163574.2
*Occludin*	F: TTGAAAGTCCACCTCCTTACAGA R: CCGGATAAAAAGAGTACGCTGG	129	NM_008756.2
*Claudin-1*	F: GGGGACAACATCGTGACCG R: AGGAGTCGAAGACTTTGCACT	100	NM_016674.4
*Bax*	F: TGAAGACAGGGGCCTTTTTG R: AATTCGCCGGAGACACTCG	140	NM_007527.4
*Bcl-2*	F: GTCGCTACCGTCGTGACTTC R: CAGACATGCACCTACCCAGC	284	NM_177410.3
*Caspase-3*	F: GGGAGCAAGTCAGTGGACTC R: GCGAGATGACATTCCAGTGC	126	NM_001284409.1

## Data Availability

Data is contained within the article. The original contributions presented in this study are included in the article. Further inquiries can be directed to the corresponding authors.
